# Sami yoik, Sami history, Sami health: a narrative review

**DOI:** 10.1080/22423982.2018.1454784

**Published:** 2018-03-26

**Authors:** Soile Hämäläinen, Frauke Musial, Anita Salamonsen, Ola Graff, Torjer A. Olsen

**Affiliations:** a National Research Center in Complementary and Alternative Medicine, Departement of Community Medicine, Faculty of Health Sciences, UiT The Arctic university of Norway, Tromsø, Norway; b RKBU North - Regional Centre for Child and Youth Mental Health and Child Welfare, Faculty of Health Sciences at UiT the Arctic University of Norway, Tromsø, Norway; c Department of Cultural Sciences, The University Museum of Tromsø, UiT The Arctic university of Norway, Tromsø, Norway; d Centre for Sami Studies (SESAM), Faculty of Humanities, Social Sciences and Education, UiT The Arctic University of Norway, Tromsø, Norway

**Keywords:** Sami, yoik, music & health, indigenous singing, emotion regulation

## Abstract

Music as a possible health-promoting agent has attained increasing academic and scientific interest over the last decades. Nonetheless, possible connections between indigenous singing traditions and health beyond traditional ceremonial healing practices are still under-researched worldwide.

The Sami, the indigenous people living in Northern Fennoscandia, have a distinct ancient vocal music tradition called “yoik” practiced from immemorial times. The Sami share a history of assimilation with many indigenous people. During this period of nearly 400 years, yoik alongside other cultural markers was under hard pressure and even banned at times.

Compared to other indigenous people in the Arctic, Sami public health shows few significant unfavourable differences to the majority population. The potential role of yoik as a protective health and resilience factor within the Sami culture is the topic of this review. We suggest a two stage model for the health promoting effects of yoik through i) ***emotion regulation and stress relief*** on the level of the individual, and ii) as a ***socio-cultural resilience factors*** within the Sami population. This review is to be understood as theory-building review article striving for a scholarly review of the literature.

## Introduction

A characteristic cultural feature of the Sami, the indigenous people living in Fennoscandia [1], is its form of singing, the yoik [1]. Yoik has particular vocal characteristics, an ancient history, and is still a living tradition within the Sami culture. What makes yoik distinct at least in some Sami-dominated areas is that it is practiced in many situations as part of everyday life. Since everything can be expressed as a yoik, people may accompany their daily activities with it. Yoik as traditional singing is thus a part of everyday life.

The survival of yoik through the centuries despite acculturation and assimilation pressures is in itself a remarkable phenomenon. Therefore we hypothesise that there may be an apparent benefit of yoik and yoik practice within the Sami culture that has protected this cultural marker from extinction. Compared to other indigenous people in the Arctic, Sami public health shows few significant differences to the majority population. Therefore, we assumed that yoik may have had and possibly still has a role as a health promoting and/or resilience factor within the Sami culture.

Music, as a particular human entity, has been used throughout times mostly in everyday life to express or alter moods, feelings and emotions [2]. Singing as such can be seen as a fundamental form of self-expression and self-regulation, containing both individual as well as social aspects [3]. Traditionally peoples all over the world sing or have songs accompanying different activities, moods, life events and occasions [4].

Music therapy (MT), as the clinically applied form of music, embraces a wide range of methods including different forms of singing [5]. During the last decade MT has build up an evidence base revealing promising indications of music’s curative potential in neurological as well as psychological disorders [6–11]. Recently the role of music in a wider health perspective of health promotion, that is music as a salutogenic agent, has been widely acknowledged. This development resulted in a new scholarly discipline called “Music and Health” (MH). In addition to traditional music therapy issues, MH focuses specifically on how music may be used to increase wellbeing and quality of life [12–14].

A systematic literature search in medical or musical databases on “Yoik/Joik/jojk/sami singing and health” in 2015 and 2016 revealed no results. This does not mean that the significance of yoik as a potential health promoting factor has been unnoticed in academic writing, but in most health related research reports this hypothesis has been discussed within a broader context of culturally important activities or contents of life [15–18]. To our knowledge, there is only one qualitative pilot study [19] directly targeting the question, whether actively practicing or passively listening to yoik can have health-promoting effects and could thus be a cultural resilience factor within the Sami culture. Due to the distinct features of yoik, which can and is often practiced without the use of words, directly expressing feelings and describing its subject with musical means, we hypothesise that a potential mechanism of action maybe through emotion regulation and the feeling of belonging. The aim of this paper is to review the accessible literature in order to build up a body of evidence supporting this theory.

## Material and methods

Because the body of knowledge specifically on yoik and health is so limited, we support our theory with evidence of possible benefits of singing to health, literature describing yoik and its possible functions, and evidence on factors that promote and support health. We present studies of Sami public health and a short overview on Sami history to illustrate the complexity of the topic.

Our main sources of references are international medical and musical library databases PubMed, ScienceDirect, Web of Science and JStor and Norwegian databases for university libraries. We used search words “Yoik/Joik/jojk/sami singing and health”, “indigenous singing/music and health”, “music and emotions”. Inclusion criteria for yoik was works concerning yoik as music. Nonetheless, the topic turned out to be too heterogeneous and complex and possibly not clinical enough for a systematic or scoping review. Therefore this review is to be understood as theory building Review article striving for a scholarly review of the literature. Moreover, these results are integrated in well established knowledge and theories from stress research, behavioral medicine and health psychology.

## The Sami

Sami is a common name for groups of indigenous people with closely related languages and cultural features, living mainly in northern parts of Norway, Sweden, Finland and Russia. Lifestyles and traditions connected to hunting, fishing, reindeer husbandry and respectively related craftsmanship are often associated with Sami culture [20]. Today less than 10% of individuals with Sami heredity are actively pursuing these traditional practices [20].

This paper focuses on Sami living in Norway, because Norway hosts the greatest part—about 70%—of the total known Sami population of the four countries [21]. Consequently, most of the public health research related to Sami heredity is done among the Sami living in Norway [22]. “The Sami living in Norway” consist of different groups like South Sami, Lule Sami, North Sami and Skolt Sami. This division is based on linguistic and geographical differences and contains of course several other features connected to culture, traditions and places [1]. The Sami were acculturated in the aforementioned countries through an assimilation politics using educational and other public systems. A substantial number Sami children were placed in boarding schools, often miles away from their homes. The purpose of these measure was assimilate these children to the dominating culture by means of education [23–26]. These children, whose native language was Sami, had to learn to speak and learn in Norwegian and refrain from their native language [21].

The most intense period of acculturation in Norway was from 1851 to 1959. In this period the idea of “Norwegianisation” was a leading ideology based on social-Darwinistic ideas of ethnic Norwegians’ racial superiority, and the importance of nation building with monocultural norms [27–31]. The latter was particularly important in the rebuilding period after WWII [28,29]. Today the majority of Sami live well integrated life as part of the Norwegian society. This is also the case for those who are self-identified as Sami and choose to participate in Sami culture and politics [32].

Currently the revitalisation of the Sami culture has grown strong and the Sami traditions have been reinvigorated by an increasing awareness and conscious efforts to preserve the Sami culture as a unique and valued part of the Norwegian society. With the foundation of the Sami Parliament of Norway 1989 [33] and the introduction of Sami as the third official language in Norway this process has had visible success. Nonetheless, the revitalising process is by no means concluded and this is particularly true regarding unsolved issues such as the utilisation of natural resources within the traditional Sami geographical regions. Conflicts between mining and reindeer herding, small- and large scale fishery, and other industrial enterprises raise fundamental questions about the rights and ownership of water and land [30].

## Sami health studies

The history of Sami is to a certain extent similar to the histories of other indigenous people including colonisation, acculturation and marginalisation through forced assimilation and discrimination. The consequences of this history commonly observed in indigenous populations are high rates of suicide, unemployment, substance abuse, low socio-economic status, and somatic diseases like CVD, diabetes, obesity, cancer and early mortality [21].

Sami public health has been subject to systematic long-term investigation for example in the SAMINOR-programme conducted in Norway in 2003–2004 and 2012–2014. This programme covered physical, psychological and health service issues of Sami living in different areas in Norway [34]. The SAMINOR-programme was preceded by the Finnmark study conducted in 1974–2000 which included the whole population of Finnmark—the Sami, Norwegians, Kven, and any others who live in the area [35]. Before these studies only some isolated reports from physicians working in North-Sami areas are available, reporting issues like tuberculosis, echinococcus and high rates of infant mortality [36–38].

Compared to other indigenous people in the arctic circumpolar area, evidence based on data from SAMINOR shows that the Sami living in Norway do not reveal significant differences in most of their public health markers and self-reported health compared to the majority population [21,39,40]. However, the Sami do differ from the majority population in that Sami women have higher adult obesity rates in Sami majority areas, and the prevalence of diabetes is higher among Sami in Southern regions where they are a minority [41]. Considerable differences are reported in self-reported cardiovascular diseases between Sami and non-Sami in northern municipalities [42].

Some studies suggest that the *similarity* in the frequency of lifestyle-related health challenges in Sami and majority population might be a result of assimilation and acculturation, because transition into the culture’s lifestyles may be followed by an increase in the prevalence of related diseases like CVD, diabetes, obesity and cancer [40,43]. Eliassen and colleagues [42] discuss that these diseases may also be understood as caused by chronic stress related to assimilation politics. According to Hansen [21], chronic stress due to ethnic discrimination in areas where Sami are a minority could be a source of a wide range of chronic diseases. Moreover, Samis are to a higher degree than the majority population disposed to violence, ethnic discrimination and bullying [44,45]. Thus, it is unclear, whether the similarities between the Sami and the majority population are a consequence of similar lifestyle, leading to a similar prevalence of lifestyle related diseases, or the consequences of acculturation pressure, which in consequence leads to a similar disease rate, despite that fact that the Sami culture may include inherent resilience factors with regard to public health.

Nonetheless, there are also caveats regarding the interpretation of Sami health studies: i) The investigation of Sami public health maybe hampered due to low participation rate in some communities [42], ii) public health surveys might not reach all individuals who might be defined as Sami descendants, because a person has to self-identify as Sami. In particular the North-South migration wave as a result of the burned earth politics of the retreating German troops in World War II known as the “burning of Finnmark”, has scattered people of Sami heritage over the whole of Norway. Many of those who came as refugees to the south decided to not identify themselves as Sami [21,28–30,45–49]. As a consequence, persons of Sami descend are possibly living everywhere in Norway even though they are not aware of it.

As a conclusion, there are no major relevant differences between the Sami population and the main population in Norway. Whether this fact is a consequence of a common lifestyle or other reasons remains unclear.

## Yoik and its history

Yoik, the singing tradition of Sami, is considered to be ancient with roots presumably in prehistoric times [23,50]. It is unique to the Sami culture and particular among European singing traditions [50]. This singing tradition is characterised by a special vocal technology that utilises nearly the whole range of the human natural vocal potential [51] and was originally without instrumental accompaniment. Use of words could vary from one region to another, from nearly none in North Sami language area to long epic descriptions in East Sami in Kola Peninsula. The melodies with regular rhythmic and melodic patterns could often be freely played with and improvised on [1,23,50,52]. Additionally, yoik could also be applied to story-telling [24,53].

The most commonly referred form of yoik is a direct, vocal musical expression of anything at the yoiker’s perception at the given moment, therefore emotions, landscapes, animals, birds and other people are yoiked [23]. Yoik often accompanies daily living and moods continuously similar to a “sonic painting” of the yoiker’s mind. Or as Buljo [23] writes: “The Sami yoik everything that belongs to human life”. According to yoikers you do not yoik about something, you yoik the thing itself [17,52]. Therefore yoik can be seen as a kind of “act of creation” as well as self-expression. It can be a way of recalling something or someone or of giving a musical name to something or someone. When missing a person or a place, yoiking the yoik belonging to the missed object or person gives the yoiker the feeling of it [17,23,53]. Therefore, unlike tone-painting, yoikers regard yoiking as a direct communication with the innermost being of the object/subject being yoiked [17,20].

Historically yoik was also used as part of shamanistic healing rituals and was a means of achieving an altered state of consciousness [23,54]. It was most likely therefore that yoik along with other pre-christian cultural elements and customs was banned as “sorcery” during the early 17th century. The Christian missionaries associated yoik with pre-christian heathen ceremonies and condemned them as “serving the Devil” [54]. Death penalty as punishment for yoiking ended first at the beginning of the 18th century. However, “sorcery” and yoik as part of it was still prosecuted [24,55,56].

Originally yoik was practiced in different forms all over the Sami-inhabited area [24]. However, during the assimilation process of the Sami culture yoik as well as many other traditional cultural features of the Sami were fundamentally weakened and had seemingly disappeared in some regions [20,47]. Moreover the societal and cultural development of what we call “modernity”, that is transition from rural to industrial-technological culture [42], has likely changed the extent to which yoik is used [20].

However, yoik is going through a fundamental revival as a musical expression. It is taught in kindergartens, schools, high schools and universities, weekend workshops and, not least, festivals [1,19]. Not only that yoik has survived a history of assimilation pressure as an element of vocal expression and communication in everyday life for many Sami, it has evolved to new forms and in fusion with different musical styles [1]. Alongside the current revival of the Sami culture, especially the younger generation finds yoik anew as a marker of identity and belonging.

## Singing, emotion regulation and well-being

Singing is for humans worldwide a way to celebrate life and death, to sooth sorrow and pain, to agitate to work as well as to war, to describe feelings and to tell stories. Singing has its place in spiritual and religious contexts in prayers, hymns and praise of the objects of beliefs. Singing traditions of many nations reveal that singing has had a natural function as a description and expression of the ongoing activity, situation or mood of the singing person.

Singing as practiced in Western cultures has received increasing scientific interest during the last decades. Several studies indicate a connection between singing as a means of self-expression, regulation of emotions, and health and well-being [57–59]. Moreover, the availability of psychobiological methodology over the last decades such as the ambulatory assessment of humoral stress markers or functional imaging have confirmed that the experienced stress releasing effects of music have indeed a psychophysiological bases [60,61]. As Hou in his review elucidates, music-evoked emotions can modulate activities in both cortical and subcortical systems, and across cortical-subcortical networks [61]. Exactly these networks, which extend from the anterior cingulate gyrus to the amygdala are integral and essential to the generation and regulation of emotions. It is particularly interesting that these networks exhibit disturbed functioning in mental and emotional psychiatric disorders.

Nonetheless, the potentially beneficial effects of indigenous singing traditions have to our knowledge predominantly been investigated as an integral part of healing rituals and here in particular within Native American contexts [62,63]. Vocal practices are understood as a natural and self-evident part of these of traditional healing methods and enhance the efficiency [62–64].

In a recent qualitative pilot study on the topic “Yoik and health” conducted in 2015, Hämäläinen and colleagues hypothesise that yoik must be considered an important marker of social and cultural belonging for many Sami people and, moreover, that it may contribute to emotion management [19]. The authors argue that yoik presents a means for a direct and non-cognitive expression of emotions [19]. It is thus different from talking about or discussing feelings, and is in this way rather similar to art related expressions of emotions such as for example painting. An important distinction to other arts is that yoik in some Sami population groups is an inherent part of everyday life, practiced continuously, accessible to everybody, and introduced during the first day of life. It might be interesting in this context, that the singing traditions of for example Inuit of Greenland and Native Americans have many functional similarities with those of the Sami [64–67].

In summary, the fact that singing can promote emotion regulation and stress release and can thus contribute to mental health and possibly beyond that to general health issues is reasonably well documented. Indigenous forms of singing have rarely been investigated with regard to their general health promoting effects, even though their impact within healing rituals is well documented. Preliminary results suggest that the way yoik is practiced where it is still practiced in the traditional way, namely as an integral part of everyday life, makes this form of singing a potential means of emotion regulation and stress relief for individuals who practice it.

## Allostatic load and resilience

Both positive and negative emotions correspond to physiological changes. This connection is mostly known through stress and the fight-flight response, however, all emotions including positive ones, have a physiological component to them [68,69]. According to Antonovsky [70] any emotion whether positive or negative, maintained over time, represents a load to human organism if not acknowledged and handled somehow, such as by adequate expression. Therefore, emotion regulation is necessary for human homeostasis, that is physiological balance and feeling of wellbeing, and an important component in human self-regulation competence [70].

Antonovsky introduced the term “salutogenesis” as a covering concept for factors supporting the maintainance and/or restitution of health. In a salutogenetic perspective, the questions of what causes disease (pathogenesis) is of lesser importance than the question of what maintains health (salutogenesis) [70]. As early as in the 1970s James P. Henry postulated a direct connection between coronary heart disease and chronic stress [71–73]. Henry’s original hypotheses have been modified considerably e.g. to the framework of the allostatic load model which describes the health impact of stress and stressful/traumatic events over the life span [74–76]. However, the fundamental assumption that chronic stress has the potential to lead to chronic disease, or at least has a negative impact on the course of chronic disease, is generally accepted. The role of trauma and especially collective trauma has received wide interest, in particular in connection with the allostatic load model. This model predicts that it is the accumulation of stressful events that may lead to a higher vulnerability for disease, such as Alzheimers disease in old age [74,76].

Significant connections have been found between disadvantageous historical factors like discrimination and assimilation pressure and unfavourable public health factors such as high rates of suicide, unemployment, substance abuse, low socio-economic status, and somatic diseases like CVD, diabetes, obesity, cancer and early mortality in indigenous populations [21,40]. However, compared to other indigenous populations, these public health marker rates are better in the total population of the Sami living in Norway. Where Sami differ to the worse from the majority population in Norway is in their disposition to discrimination, bullying and violence [21,44]. Nonetheless, social support within a group, in particular a cultural group, constitutes a significant socio-cultural resilience factor, as has been established for example Holocaust survivors. Social support can provide a strong protective factor against the long-term consequences of trauma [70,77]. Insofar is the hypothesis that belonging to a specific cultural subgroup may have served as e protective factor evident.

With regard to the potential role of yoik as a cultural resilience factor Bals and colleagues report participation in traditional and cultural activities being a significant protective factor for Sami youth mental health [15]. Moreover, in a study of cultural resilience factors among Sami adolescents living in a Sami community, personal yoik was established as being such a factor [16]. Hanssen has presented some interesting findings suggesting a possible significance of yoik as a valuable cultural symbol in intercultural health care communication with elderly Sami patients in nursing homes [17,18]. Moreover, Hämäläinen et al. conclude that yoik serves as a cultural resilience factor by being an important marker of social and cultural belonging and an instrument for emotion management on an individual bases [19].

In summary, in the light of the context of salutogenesis, yoik may support individual health through emotion regulation and stress relief on the basis of the person. Beyond that, the fact that yoik represents a fundamental marker of socio-cultural belonging makes this particular form of indigenous singing likely an element of cultural resilience within a population based perspective.

## Conclusion

The aim of this review was to investigate the role of yoik as a potential health promoting factor within the Sami culture. After reviewing the available evidence, we suggest a two-step model for how yoik can promote health:

### Individual level

Yoik serves as a means of ***emotion regulation and stress relief*** on the level of the individual. To actively yoik and to listen to yoik evokes positive emotions and feelings of belonging. These have been established as an important factor promoting salutogenesis.

### Population level

Yoik is a significant cultural marker who has survived throughout centuries, even though it was heavily prosecuted. Cultural markers serve as symbols of identity and belonging. Such symbols in a particular a cultural group, can constitute powerful ***socio-cultural resilience factors***.

The role of positive emotions, optimism and belonging/social support as health protective factors has long been established. Even though many of these results are derived from severely traumatised groups, such as holocaust survivors, the accumulated knowledge has nonetheless general and fundamental significance for our understanding of resilience [5–7]. The role of positive emotions such as love and the social bond for human and animal well-being has meanwhile been accepted even in neuroscience [8]. What is less known is the acknowledgement of music as a related human expression. Jaak Panksepp, the pioneer of affective neuroscience describes in his hallmark publication, Affective Neuroscience—The foundation of human and animal emotions, the role of music as such: “That audiovocal experience speaks to us of our humaneness and our profound relatedness to other people and the rest of nature” [9]. It is striking how much this understanding of music resembles the meaning of yoik expressed by many yoikers. What could confirm the neuroscientist’s perspective better than the statement of a female yoiker Biret Risten Sara from Northern Norway: “Where words end and become insufficient to express the depth of the experience, that is where yoik begins” [78].

## References

[1] Hilder T. Sámi musical performance and the politics of indigeneity in Northern Europe. Anham(MD): Rowman & Littlefield; 2015.

[2] Thoma MV, Ryf S, Mohiyeddini C, et al Emotion regulation through listening to music in everyday situations. Cogn Emotion. 2012;26(3):550–8. 10.1080/02699931.2011.595390 21902567

[3] Juslin P, Sloboda J Handbook of music and emotion: theory, research, applications. Oxford: Oxford University Press; 2010.

[4] Theorell T Psychological health effects of musical experiences. Theories, studies and reflections in music health science. Dordrecht, Heidelberg, New York, London: Springer; 2014.

[5] American Music Therapy Association (AMTA). What is music therapy?; 2018 Available from: https://www.musictherapy.org/

[6] Kamioka H, Tsutani K, Yamada M, *et al* Effectiveness of music therapy: a summary of systematic reviews based on randomized controlled trials of music interventions. Patient Prefer Adherence. 2014;8:727–754. 24876768 10.2147/PPA.S61340 PMC4036702

[7] Moore KS A systematic review on the neural effects of music on emotion regulation: implications for music therapy practice. J Music Ther. 2013;50(3):198–242. 24568004 10.1093/jmt/50.3.198

[8] Myskja A Integrated music in nursing homes: an approach to dementia care. Bergen: Universitetet i Bergen; 2012.

[9] Särkämö T, Tervaniemi M, Laitinen S, et al Cognitive, emotional, and social benefits of regular musical activities in early dementia: randomized controlled study. Gerontologist. 2014;54(4):634–650. 24009169 10.1093/geront/gnt100

[10] Kvamme TS Glimt av glede. Musikkterapi med demensrammede som har symptomer på depresjon og angst. Oslo: Monograph; 2013.

[11] Barnish J, Atkinson RA, Barran SM, et al Potential benefit of singing for people with Parkinson’s disease: a systematic review. J Parkinson’s Dis. 2016;6:473–484. 27258698 10.3233/JPD-160837

[12] CREMAH. Norwegian Academy of Music, Centre for Research in Music and Health; 2018 Available from: https://nmh.no/en/research/cremah

[13] Novotney A Music as medicine. Monitor Psychol. 2013;44(10):46.

[14] MacDonald R Music, health, and well-being: A review. Int J Qualitative Stud Health Well-Being. 2013;8(1):20635. 10.3402/qhw.v8i0.20635 PMC3740599 23930991

[15] Bals M, Turi AL, Skre I, et al The relationship between internalizing and externalizing symptoms and cultural resilience factors in Indigenous Sami youth from Arctic Norway. Int J Circumpolar Health. 2011;70(1):37–45. 21329576 10.3402/ijch.v70i1.17790

[16] Nystad K, Spein AR, Ingstad B Community resilience factors among indigenous Sámi adolescents: a qualitative study in Northern Norway. Transcultural Psychiatry. 2014;51(5):651–672. 24846701 10.1177/1363461514532511

[17] Hanssen I A song of identity: yoik as example of the importance of symbolic cultural expression in intercultural communication/health care. J Intercultural Commun. 2011;27:ISSN: 1404–1634.

[18] Hanssen I The influence of cultural background in intercultural dementia care: exemplified by Sami patients. Scand J of Caring Sci. 2013;27(2): s231–237. DOI: 10.1111/j.1471-6712.2012.01021.x2013. 10.1111/j.1471-6712.2012.01021.x 22686451

[19] Hämäläinen S, Musial F, Graff O, et al Yoik experiences and possible positive health outcomes: an explorative pilot study. Int J Circumpolar Health. 2017;76(1):1271590. 10.1080/22423982.2016.1271590 PMC5328371 28452679

[20] Jones-Bamman RW “As long as we continue to joik, we’ll remember who we are”: negotiating identity and the performance of culture: the Saami joik. Seattle (WA): University of Washington; 1993.

[21] Hansen KL Ethnic discrimination and health: the relationship between experienced ethnic discrimination and multiple health domains in Norway’s rural Sami population. Int J Circumpolar Health. 2015;74(1):25125. 10.3402/ijch.v74.25125 PMC4329315 25683064

[22] Stoor P Kunskapssammanställning om samers psykosociala ohälsa. Giron/Kiruna: Sametinget; 2016.

[23] Buljo KA Samisk musikk In: Aksdal B, Buljo KA, Fliflet A, et al, editors. *Trollstilt: lærebok i tradisjonsmusikk* edn. Oslo: Gyldendal undervisning; 1998 p. 137–160.

[24] Graff O “Om kjæresten min vil jeg joike”: undersøkelser over en utdødd sjøsamisk joiketradisjon. Karasjok: Davvi girji; 2004.

[25] Graff O Joikeforbudet i Kautokeino. Karasjok: Davvi girji; 2016.

[26] Hætta OM Samene: nordkalottens urfolk. Kristiansand: Høyskoleforlaget; 2002.

[27] Pedersen S Samiske sedvaner og rettsoppfatninger – bakgrunnsmateriale for samerettsutvalget. Rapporter fra et forskningsprosjekt om samiske sedvaner og rettsoppfatninger, ledet av en styringsgruppe oppnevnt 15. mai 1996 In: beredskapsdepartementet J-o, editor, 2001 ed., Vol. 34 Oslo: Den Norske regjeringen Statens forvaltningstjeneste Informasjonsforvaltning; 2001 Available from: https://www.regjeringen.no/no/dokumenter/nou-2001-34/id379485/sec1.

[28] Bjørklund I Fjordfolket i Kvænangen: fra samisk samfunn til norsk utkant 1550–1980. Tromsø: Universitetsforlaget; 1985.

[29] Eriksen K, Niemi E Den finske fare: sikkerhetsproblemer og minoritetspolitikk i nord 1860–1940. Oslo: Universitetsforlaget c; 1981.

[30] Pedersen P, Høgmo A Sápmi slår tilbake: samiske revitaliserings- og moderniseringsprosser i siste generasjon. Kárášjohka: ČálliidLágádus; 2012.

[31] Minde H Assimilation of the Sami: implementation and consequences. *Galdu Cala* C. 2005;3 Available from: http://galdu.custompublish.com/getfile.php/3307993.2388.dvvstfwfxw/mindeengelsk.

[32] Pettersen T, Brustad M Which Sa´mi? Sa´mi inclusion criteria in population-based studies of Sa´mi health and living conditions in Norway an exploratory study exemplified with data from the SAMINOR study. Int J Circumpolar Health. 2013;72(1):21813. 10.3402/ijch.v72i0.21813 PMC3838972 24282785

[33] The Sami Parliament in Norway; 2018 Available from: https://www.sametinget.no/Om-Sametinget/Bakgrunn

[34] SAMINOR. UiT The Arctic University of Norway, Centre for Sami Health Research; 2018 Available from: https://en.uit.no/forskning/forskningsgrupper/gruppe?p_document_id=425187

[35] Forsdahl A, Fylkesned K, Hermansen R, et al Hjerte-karundersøkelsene i Finnmark 1974–2000: resultater fra undersøkelsene. ISM Skriftserie. 2001;58:ISBN : 8290262655.

[36] Berg S Finnemisjonen eller Samemisjonen i Norge. Trondhjem: Norges Finnemisjonsselskab; 1926.

[37] Rein K Spedbarnsdødeligheten i Kautokeino 1946–1955. Tidsskrift for Den Norske Lægeforening. 1956;76(21):815–816. 13380863

[38] Myrseth Echinokokksykdommen i Finnmark. Tidsskrift for Den Norske Lægeforening. 1956;76(22):867–871. 13380871

[39] Anderson I, Robson B, Connolly M, *et al* Indigenous and tribal peoples’ health (The lancet–lowitja institute global collaboration): a population study. The Lancet. 2016;388(10040):131–157. 10.1016/S0140-6736(16)00345-7 27108232

[40] Eliassen BM, Braaten T, Melhus M, et al Acculturation and self-rated health among Arctic indigenous peoples: a population based cross-sectional study. BMC Public Health. 2012;12:948. 10.1186/1471-2458-12-948 PMC3497910 23127197

[41] Naseribafrouei AE A, Eliassen M, Melhus M, et al Ethnic difference in the prevalence of pre-diabetes and diabetes mellitus in regions with sami and non-sami populations in Norway – the SAMINOR1 study. Int J Circumpolar Health. 2016;75:31697. 10.3402/ijch.v75.31697 PMC4978855 27507149

[42] Eliassen BM, Melhus M, Hansen KL, et al Marginalization and cardiovascular disease among rural Sami in Northern Norway: a population-based cross-sectional study. BMC Public Health. 2013;13(522). 10.1186/1471-2458-13-522 PMC3668238 23718264

[43] Sjölander P What is known about the health and living conditions of the indigenous people of northern Scandinavia, the Sami? Global Health Action. 2011;4. 10.3402/gha.v4i0.8457 PMC3195409 22007156

[44] Eriksen AMA, Hansen KL, Javo C, et al Emotional, physical and sexual violence among Sami and non-Sami populations in Norway: the SAMINOR 2 questionnaire study. Scand J Public Health. 2015;43:588–596. 25969164 10.1177/1403494815585936

[45] Hansen KL, Melhus M, Høgmo A, et al Ethnic discrimination and bullying in the Sami and non-Sami populations in Norway: the SAMINOR study. Int J Circumpolar Health. 2008;67(1):97–113. 18468262

[46] Eidheim H Aspects of the Lappish minority situation, 3rd ed. Oslo: Universitetsforlaget; 1977.

[47] Grenersen G Ved forskningens grenser. Historien om et forskningsprosjekt i det samiske nord-norge. Oslo: Spartacus Forlag AS; 2002.

[48] Minde G-T, Sæterstrand TM What is important in the surroundings in order to extend the healthy life period? A regional study of 19 older women in a northern part of Norway. Int J Circumpolar Health. 2013;72(1):1–6. 10.3402/ijch.v72i0.21189 PMC3749851 23971013

[49] Pettersen T, Brustad M Same Sámi? a comparison of self-reported Sámi ethnicity measures in 1970 and 2003 in selected rural areas in northern Norway. Ethn Racial Stud. 2015;38:2071–2089.

[50] Szomjas-Schiffert G Traditional singing style of the lapps. Yearbook of the International Folk Music Council. 1973;5:51–61.

[51] Daling G Joik og stemmebruk: en beskrivelse av joik med komplett sangteknikk s om innfallsvinkel In: Johansen KL, Edited by. *Slik vi ser det: om musikk og musikkundervisning sett med elleve par høyskoleøyne* edn. Levanger: Musikkseksjonen ved Høgskolen i Nord-Trøndelag; 2014 p. 158–165.

[52] Edström O From Jojk to Rock & Jojk: some remarks on the process of change and of the socially constructed meaning of sami music. Studia Musicologica Academiae Scientiarum Hungaricae. 2003;44(1/2):269–289.

[53] Saastamoinen I, Graff O Son vuäinn. She sees – Skolt Sámi leu’dd from the Kola Peninsula. Helsinki: Global Music Centre; 2007.

[54] Wersland EM Joik i den gamle samiske religionen yoik in the old Sami religion. Nesbru: Vett & viten; 2006.

[55] Gaski H, Graff O «-Joik har større kraft enn krutt» In: Drivenes E-A, Hauan MA, Wold HA, editors. *Nordnorsk kulturhistorie*. edn. Oslo: Gyldendal; 1994 p. 403–413.

[56] Rydving H The end of drum-time. Religious change among the Lule Saami 1670s–1740s, 3rd ed. Uppsala: Uppsala Universitet; 2004.

[57] Clift SM, Hancox G The perceived benefits of singing: findings from preliminary surveys of a university college choral society. J R Soc Promot Health. 2001;121(4):248–256. 11811096 10.1177/146642400112100409

[58] Wan CY, Ruber T, Hohmann A, et al The therapeutic effects of singing in neurological disorders. Music Percept. 2010;27(4):287–295. 21152359 10.1525/mp.2010.27.4.287 PMC2996848

[59] Bradt J, Dileo C, Potvin N Music for stress and anxiety reduction in coronary heart disease patients. Cochrane Database Syst Rev. 2013;12:CD006577. 10.1002/14651858.CD006577.pub3 PMC8454043 24374731

[60] Linnemann A, Ditzen B, Strahler J, et al Music listening as a means of stress reduction in daily life. Psychoneuroendocrinology. 2015;60(Supplement C):82–90. 26142566 10.1016/j.psyneuen.2015.06.008

[61] Hou J, Song B, Chen ACN, et al Review on neural correlates of emotion regulation and music: implications for emotion dysregulation. Front Psychol. 2017;8:501. 28421017 10.3389/fpsyg.2017.00501 PMC5376620

[62] Bassett D, Tsosie U, Nannauck S “Our culture is medicine”: perspectives of native healers on posttrauma recovery among American Indian and Alaska Native patients. Perm J. 2012;16:19–27. 10.7812/tpp/11-123 PMC3327107 22529755

[63] Crawford O’Brien S Religion and healing in native America: pathways for renewal. Westport, Conn: Praeger Publishers; 2008.

[64] Gioia T Healing Songs. North Carolina: Duke University Press; 2006.

[65] Bad Hand HP Native American Healing. Chicago: Keats Publishing; 2002.

[66] Hauser M Traditional greenlandic music. København: Kragen/ULO; 1992.

[67] Sjöström N Indigenous people’s intuitive use of music as therapy enhances the authenticity of music therapy today. In Museum TU editor. Tromsø: Tromsø Museum Archive; 1991.

[68] Breedlove SM, Rm R, Wn V Biological psychology: an introduction to behavioral, cognitive, and clinical neuroscience. 5th ed. Sunderland: Mass Sinauer Associates c; 2007.

[69] Choi K, Musial F Emotionen In: Paul A, Dobos G, editors. *Mind/body medicine*. Germany: Elsevier; 2011 p. 70–77.

[70] Antonovsky A Unraveling the mystery of health. How people manage stress and stay well. San Francisco (CA): Jossey-Bass Publishers; 1987.

[71] Henry J, Stephens P, Ely D Psychosocial hypertension and the defense and defeat reactions. J Hypertens. 1986;4(6):687–697. 3546493 10.1097/00004872-198612000-00002

[72] Henry J, Stephens P The social environment and essential hypertension in mice: possible role of the innervation of the adrenal cortex. Prog Brain Res. 1977;47:263–276. 928750 10.1016/S0079-6123(08)62731-4

[73] Henry JP Mechanisms by which stress can lead to coronary heart disease. Postgrad Med J. 1986;62(729):687–693. 3748938 10.1136/pgmj.62.729.687 PMC2418752

[74] McEwen BS Mood disorders and allostatic load. Biol Psychiatry. 2003;54(3):200–207. 12893096 10.1016/s0006-3223(03)00177-x

[75] McEwen BS, Stellar E Stress and the individual: mechanisms leading to disease. Arch Intern Med. 1993;153(18):2093–2101. 8379800

[76] Karlamangla AS, Singer BH, McEwen BS, et al Allostatic load as a predictor of functional decline: Macarthur studies of successful aging. J Clin Epidemiol. 2002;55(7):696–710. 12160918 10.1016/s0895-4356(02)00399-2

[77] Fossion P, Leys C, Kempenaers C, et al Psychological and socio-demographic data contributing to the resilience of holocaust survivors. J Psychol. 2014;148(6):641–657. 25175888 10.1080/00223980.2013.819793

[78] Sara BR A speech on traditional yoik. Tromsø: 2015.

